# Sex-Specific Characteristics of Perivascular Fat in Aortic Aneurysms

**DOI:** 10.3390/jcm14093071

**Published:** 2025-04-29

**Authors:** Katja Heller, Panagiotis Doukas, Christian Uhl, Alexander Gombert

**Affiliations:** Department of Vascular Surgery, RWTH Aachen University Hospital, 52074 Aachen, Germany; pdoukas@ukaachen.de (P.D.); cuhl@ukaachen.de (C.U.); agombert@ukaachen.de (A.G.)

**Keywords:** aortic aneurysm, sex differences, inflammation, perivascular adipose tissue, vascular dysfunction

## Abstract

Aortic aneurysms (AAs), the dilation or widening of the aorta, lead to dissection or rupture with high morbidity and mortality if untreated. AA displays gender disparities in its prevalence, progression and outcomes, with women having worse outcomes and faster aneurysm growth. However, current guidelines do not address gender dimorphism, emphasizing the urgent need for personalized treatment strategies and further research. Perivascular adipose tissue (PVAT), a unique type of fat surrounding blood vessels, plays a critical role in maintaining vasomotor tone and vascular homeostasis, with dysfunction associated with chronic inflammation and vessel-wall remodeling. Indeed, PVAT dysfunction promotes the development of aortic aneurysms, with hormonal and biomechanical factors exacerbating the pathological vascular microenvironment. The sexually dimorphic characteristics of PVAT include morphological, immunological, and hormonally mediated differences. Thus, targeting PVAT-mediated mechanisms may be a promising option for the (gender-specific) therapeutic management of cardiovascular pathologies. This review examines the emerging importance of PVAT in vascular health, its potential therapeutic implications for AA, and identifies gaps in the current state of research.

## 1. Introduction

An aortic aneurysm (AA) is a dilation of the aorta [1]. Risk factors include ageing, hypertension, smoking, atherosclerosis, family history and male sex. Untreated AA can lead to aortic dissection or rupture, often with high morbidity and mortality [2].

The aorta, the largest elastic artery, is divided into segments, including the ascending aorta, the aortic arch, the descending thoracic aorta, and the abdominal aorta [3]. AAs are typically classified according to their location as abdominal aortic aneurysms (AAAs) or thoracic aortic aneurysms (TAAs) [4]. Whereas TAAs are known to have a stronger genetic component in diseases like Marfan’s and Loeys-Dietz’s, AAAs are primarily linked to arteriosclerosis [5,6]. Both share common features such as a dilated aortic phenotype, loss of vascular smooth muscle cells (vSMCs), inflammation and extracellular matrix (ECM) modifications [5]. Remarkably, despite striking similarities at the gross anatomical level, the underlying pathophysiology is quite distinct [7,8], including the embryonic origin of vSMCs [9,10].

AAAs are responsible for approximately 1.3% of deaths in men aged 65 to 85 years in developed countries [7]. TAA, typically of indolent nature, often goes undetected unless found by chance, through family screening, or acute aortic events. Although less prevalent than AAA, TAA affects 6–10 individuals per 100.000 [8]. In addition, AAs exhibit marked gender disparities in clinical presentation and outcomes. While men are more frequently affected, with a male-to-female ratio of 5:1 [8], women experience significantly worse results [11]. Until now, the proof in the guidelines, such as of the European Society of Vascular Surgery (ESVS) 2024, has been based primarily on the data of men, as the number of women with AAA in all randomized trials was limited [1]. Evidence advises that women progress more rapidly with AA and face higher risks of life-threatening events, yet current referral thresholds only partially reflect gender, e.g., the ESVS recommendation to intervene at a diameter of 5 cm in women compared to 5.5 cm in men [1].

Inflammation is a critical step in the pathogenesis and progression of AA, including AAA and TAA, driven by the recruitment of inflammatory cells to the vessel wall [12,13]. Perivascular adipose tissue (PVAT), a metabolically active fat, modulates vascular tone and homeostasis via endocrine/paracrine secretion of vasoactive and inflammatory factors such as adipocytokines [14,15]. Metabolic confounders—like obesity, type 2 diabetes and atherosclerosis—impair PVAT function, promoting a pro-inflammatory, pro-oxidative and vasoconstrictive state, thereby contributing to vascular pathologies through bidirectional interactions with the cellular vessel wall [15,16,17].

PVAT is increasingly recognized as a multifunctional organ and potential therapeutic target in cardiovascular disease. The extent to which the perivascular space serves not only as a connective but also as a critical secretory compartment in the progression of vascular diseases, such as aortic aneurysm, is still being explored. Studying the highly adaptive PVAT may help to develop targeted interventions. Specifically, exploring the sex-specific nature of PVAT is crucial for identifying the underlying causes of sex-based disparities in disease outcomes. To advance personalized approaches for the prevention and treatment of cardiovascular disease, further research into molecular and metabolic factors that influence such variation is mandatory.

## 2. Gender-Specific Differences in Aortic Aneurysms

Gender bias in the management and outcomes of AAA has long been recognized, with apparent differences in prevalence, clinical presentation and endpoints. Women tend to suffer worse outcomes, including higher mortality rates following aortic repair, both perioperatively and long-term, whether endovascular or open surgery [18,19,20,21].

A 1997 retrospective study of diagnosed AAAs showed that women not only underwent less aortic reconstruction, but had significantly lower survival [18]. Consistent with a pattern of Beller et al., 2015, which discovered a higher 30-day mortality in women after ascending AA [22]. Recently, Jiang et al., 2024 revealed that the outcome after TAA surgery in a total of 455 patients was less favorable in women with a significantly prolonged overall mortality of 11% compared to 4.9% [19]. Female gender has already been shown to be a decisive risk factor, with women suffering from AAA having a higher risk of developing a concomitant TAA [21]. Moreover, women with AAAs face up to a 4-fold increased risk of rupture [11,23], although they typically exhibit smaller diameters [24]. Plus, women die of ruptured AAAs at an older age [25]. In 2024, Notenboom and colleagues published the DisSEXion study, which included 1850 patients, to determine the differences between men and women regarding aneurysm growth and clinical outcomes. Apart from similar maximum aortic diameters at diagnosis, women typically experience faster aneurysm growth, particularly in the ascending aorta, and have a higher incidence of type A dissections, 3.4 times more frequent than in men. The growth pattern is distinct, with a faster tubular ascending growth in females, which may contribute to the increased risk of rupture [26]. In consequence, using a similar AAA size threshold for elective surgery for men and women may not be appropriate.

### 2.1. Hormonal and Biomechanical Factors

The underlying causes for sexual dimorphism in vascular pathologies are manifold. Hormonal imbalance, particularly those associated with the menopause, is a major contributing factor. Estrogen has been thought to exert vasculoprotective properties in women [27], whereby its postmenopausal decline has been associated with increased endothelial dysfunction, arterial stiffening and accelerated cardiovascular risk [11,28]. In men, progesterone and estradiol measured in serum are inversely associated with AAA [29], pointing to a connection between steroids and AAA formation. Strikingly, higher levels of estradiol and progesterone have been reported in men with AAA in another prospective case-control study [30], highlighting the inconclusive nature of current evidence. This heterogeneity is also echoed in the clinical trials of women on hormone replacement therapy, as reviewed by Makrygiannis et al. [31]. In addition to estrogen, androgens may interfere with AAA formation by affecting the renin–angiotensin pathway and their ability to promote ECM remodeling through upregulation of MMP2 [32]. Thus, experimental orchidectomy in mice significantly reduced the incidence of AAA in males as opposed to females, with an increase in AAA in both groups treated with dihydrotestosterone. Surprisingly, ovariectomy did not affect AAA formation in mice, according to the same study [32]. Supporting this, Alsiraj and colleagues noted that testosterone promoted rupturing in female mice, accompanied by elevated inflammation, proteolytic activity, and oxidative stress [33]. A substitute animal study suggested a mechanism by which androgens exacerbate AA by suppressing the expression of cell death protein−1 in T-cells [34]. However, whether androgens protect or deteriorate AA remains elusive. Despite the idea of an adverse androgen action, e.g., in older men, as some data associate lower testosterone levels with AAA [35]. A meta-analysis of 70 studies similarly revealed that patients with cardiovascular complications tended to have lower levels of testosterone [36], further confounding the interpretation. In addition to hormonal factors, biomechanical disparities occur, with postmenopausal women experiencing increased aortic stiffness, exacerbated by smoking, regardless of the lessened elasticity and collagen content of female aortic tissue per se [37]. Both post- and premature menopause are predictors of AAA in women with a substantial smoking history [38], possibly due to an increased protease activity. Indeed, greater MMP expression in females with AAA make it an important contributor to AAA pathogenesis [11,28,37,39]. As such, the increased growth rate and risk of rupture in women may be explained by differing levels of protease expression, even at comparable aortic diameters [11].

### 2.2. Gender Bias in Treatment and Surgical Outcomes

Despite the urgency of these findings, current aortic disease guidelines do not adequately address gender differences in terms of progression rates, treatment thresholds, and long-term prognosis [1]. Until now, no formal consensus has been reached on the exact pathological background and whether men and women with AAA should be treated differently. Research is needed to ensure gender-sensitive approaches to the onset, presentation and outcome of AAA.

## 3. Perivascular Adipose Tissue

### 3.1. Anatomy and Structural Composition

Perivascular adipose tissue (PVAT) is a specialized type of adipose tissue that envelops large blood vessels, playing a central role in maintaining the vascular microenvironment. PVAT consists of a variety of cell subtypes including 40–50% adipocytes surrounded by fibrous connective tissue, nerves, an extensive vasa vasorum and stromal cells—which collectively contribute to its unique physiological function. The stromal-vascular fraction includes preadipocytes, fibroblasts, endothelial cells, mesenchymal cells, vSMC and immune cells [40,41,42]. The highly adaptive morphology of PVATs exhibits considerable heterogeneity, displaying characteristics of brown and white adipose tissue, with different developmental, functional and secretory properties [43,44]. The distinctive plasticity is particularly dependent on the anatomical location, e.g., in proximity to the coronary, thoracic or abdominal artery. In contrast to thoracic fat, which resembles thermogenic brown adipocytes, abdominal periaortic vasculature has features similar to metabolic white cells [41,45]. While clear differences between PVAT types are supported by evidence, most studies have been conducted in rodents, and less is known about human PVAT, which primarily consists of white adipocytes [41,46]. The aspects described are listed in Table 1.

### 3.2. Normal and Pathological PVAT Functioning

PVAT is receiving increasing attention due to its a highly adaptive plasticity, although detailed knowledge of its various physiological and pathological properties is still rudimentary. In addition to the mechanical support function, the metabolic and secretory capabilities of the fat have been emerging [41,47].

Under normal conditions, PVAT plays a protective role in vascular health by secreting factors that regulate vascular tone and prevent inflammation, contributing to an anti-atherosclerotic environment. Among these are adipokines like leptin and adiponectin [48,49]. Various adipocytokines play a pivotal role, with both pro-inflammatory and anti-inflammatory factors coexisting to sustain vascular homeostasis. While pro-inflammatory cytokines such as TNF-α [50] and IL-6 [51] induce inflammation and endothelial dysfunction by recruiting immune cells, the anti-inflammatory IL-10 is able to improve endothelial function and nitric oxide (NO) production [49]. CD68^+^ macrophages and CD4^+^ T-cells have been found to accumulate in PVAT of obese mice, causing chronic inflammation and impaired vasodilation [52]. Adipocytokines are not the only vascular resistance players contributing to hypertension and cardiovascular disease [53,54]. Adipocyte-derived relaxation factor (ADRF) and perivascular-derived relaxation factor (PDRF) are two potent types of PVAT factors mediating vasorelaxation [55,56]. Mechanistically, perivascular stromal cells control the relaxation, proliferation and migration of vSMCs during vascular stress by sending signals from the outside to the inside of the vessel wall [55].

Altered nutrient availability affects the expression of matrix proteins and catabolic enzymes. If the matrix is unable to adapt, e.g., due to hypoxia, systemic inflammation or fibrosis, hypertrophic adaptations of cells may occur [57,58]. Under pathological conditions, mainly metabolic imbalances such as obesity and atherosclerosis, PVAT becomes malfunctional, facilitating a vasoconstrictive and pro-inflammatory microenvironment [41,46]. Bidirectional crosstalk is the interaction between PVAT and the vessel wall during plaque formation. During proatherogenic conditions, adipokines released from PVAT act as outside-in signal to drive plaque formation. This in turn leads to an altered response to local inflammation in the damaged vessel wall, described as inside-to-outside signaling [17,59]. In particular, the early phase of endothelial function impairment is marked by a drop in hydrogen sulfide, NO and adiponectin, an increase in leptin, IL-6, TNF-α, IL-12, and renin-angiotensin-aldosterone-system activation [55,60,61]. As mentioned above, adverse changes in the pattern of lipid secretion contribute to vascular oxidative stress through reduced NO availability [46], increased reactive oxygen species (ROS) production [62] and low-grade inflammation [42,63]. During plaque formation, low density lipoprotein (LDL) enriches the arterial wall, followed by inflammatory cells, the migration of vSMCs as well as foam cell formation fostered by PVAT-derived adipocytokines. The pro-inflammatory milieu downregulates collagen synthesis, thins the fibrous cap and increases plaque vulnerability [41]. The aspects described are listed in Table 2 as an overview.

## 4. PVAT and Aortic Aneurysm

The aorta is composed of three layers: intima, media and adventitia. While the inner adventitial barrier is well defined by the outer elastic media lamina, there is no clear outer adventitial demarcation, forming a continuum with PVAT [42,64,65,66]. Due to its anatomical proximity to the vascular adventitia, adaptive plasticity and its considerable secretory activity, PVAT has been implicated directly and indirectly in aortic aneurysm formation and progression. Based on a 2021 systematic review, animal and human studies examining the effect of PVAT on AA are limited, although several studies have investigated PVAT-derived adipokines [67]. To date, the relevance of the perivascular space under pathological conditions and unravelling the complexity of PVAT biology and its systemic functionalities are increasingly being discussed in the context of vascular research.

Adventitia and PVAT play key roles in vascular function by acting as receptors for substances and facilitating communication with the vascular wall [55]. Beyond its endocrine properties, fat can directly affect the paracrine or vasocrine vessel wall [17]. PVAT is thought to respond to paracrine signals from vascular cells to facilitate phenotypic changes in PVAT adipocytes and alter their secretome composition (inside-out), which in turn affects the vascular wall (outside-in) [17].

The ECM is a complex, non-cellular network of matrix elements and cell surface receptors [68,69]. The integrity of the ECM is critical for the proper functioning of various cells and organs, whereby dysfunction of the tECM composition and structure is associated with several metabolic diseases. Obesity, for example, dramatically increases the size and number of adipocytes, mechanically supported by the high activity and rapid micro-anatomical adaptability of the matrix [68,69]. Failure of the ECM to adapt results in a hypertrophic cell limit, correlating with characteristic adipocyte adaptations, leading to hypoxia, systemic inflammation or fibrosis [58,70]. Emerging evidence suggests that like states of metabolic abnormalities, the number and size of PVAT cells increase with AAA diameter [55,71]. Using microarray analysis of the PVAT of 30 patients with AAA, Piacentini et al., identified significant changes in gene expression in the aorta that were associated with local PVAT gene signature. Overrepresented genes were related to immune response, cell death pathways, collagen metabolism, sphingolipids, aminoglycans and ECM degradation in AAA PVAT [71]. Several animal and human studies provide evidence that excessive fat accumulation in the wall of AAA precipitates rupture [72,73]. The enrichment of adipocytes in AAA patients has been attributed to adipose-derived stem cells in the aneurysmal adventitia and to the expression of peroxisome proliferator-activated receptor gamma2 (PPARγ2) [73]. The protein encoded by the gene PPARγ (Pparg) is a central regulator of adipocyte differentiation. Its deletion leads to loss of adipose tissue in rodents [74]. Reduced expression of PPARγ2 is consistent with the observation that decreased levels of anti-inflammatory genes were observed in the PVAT of AAA patients, whereas pro-inflammatory genes were upregulated [75]. Interestingly, AA-mice treated with PPAR ligands (pioglitazone and fenofibrate) showed a reduced suprarenal aortic expansion and macrophage accumulation compared to the vehicle group [76]. Mechanistically, PPAR activation likely prolongs a compensatory phase of inflammatory cytokine expression, slowing AAA development and rupture [77]. This is consistent with the fact that diabetics are less prone to the development, progression and rupture of AAA [78]. Additionally, during the progression of atherosclerosis, PVAT-PPAR deficiency correlates with impaired thermogenic capacity, vascular and systemic inflammation in mice [79,80]. Of note, human preadipocytes characterized less intracellular lipid accumulation when cocultured with angiotensin II-treated aortic tissue, suggesting that inflammatory mediators prevent preadipocyte differentiation into mature adipocytes [81]. Together, reduced PPARγ expression is linked to increased inflammation and faster AAA growth, so pharmacologically activating PVAT-PPARγ may represent a promising strategy to postpone or prevent AAA. Further studies are warranted to verify the role of PVAT-mediated pro-adipogenic/anti-inflammatory markers for potential AAA risk reduction.

Moreover, macrophage infiltration, increased MMPs and reduced collagen were observed around rat aortic tissue of the ruptured cohort [72]. In fact, as evidenced by an elevated pro-inflammatory and MMP gene expression profile, human AAA patients also had exacerbated wall inflammation due to PVAT [75]. Another enrichment analysis of Piacentini et al. revealed an excess of immune, cell death, collagen metabolism, sphingolipid and ECM degradation genes in AAA PVAT, and these changes were restricted to PVAT around the aneurysm [82]. Subsequently, T-cells have been implicated as an eminent immune cell type in AAA pathogenesis. When activated, they stimulate macrophages in AAA to release pro-inflammatory substances [14]. PVAT is thought to serve as an immune cell reservoir: PVAT T cells are significantly activated, and their infiltration appears to be associated with AAA size [83,84,85,86]. Furthermore, AAA PVAT-conditioned medium upregulated CD14-dependent monocytes in mice [87].

Another growing focus is on the cellular interaction of the PVAT stroma and vSMCs. PVAT dysregulation leads to inflammation, contributing to AA progression via vascular dysfunction and remodeling. The role of vSMCs in aortic aneurysms is controversial. vSMCs contribute to vascular stability but can also promote inflammation through phenotypic switching [88,89]. PVAT inflammatory signaling, secretion of growth factors and disruption of the local vSMC behavior have all been implicated in the weakening of vessel walls leading to aneurysm formation [14]. In this context, PVAT-derived cytokine-dependent mechanisms (e.g., monocyte chemoattractant protein-1, C-reactive protein), have been implicated in the pathogenic effects of aneurysm formation, including inflammation, vSMC proliferation and neointimal hyperplasia [14]. Moreover, PVAT-derived adipokines such as leptin may stimulate vSMC remodeling via the p38 MAPK-dependent pathway [90,91,92,93,94]. Furthermore, PVAT-derived adiponectin partially compensates for age-related decline in NO-mediated vasodilation in SMC-knockout rat thoracic aortas [95]. Observations by Huang et al. offer a potential approach for AAA therapy by demonstrating the antiapoptotic effects of thoracic PVAT on vSMCs, resulting in reduced AAA formation, likely due to the secretome of browning adipocytes [96]. Overall, PVAT affects vSMCs in AA by balancing inflammatory and protective factors, opening up the possibility of stabilizing the vessel wall.

Retrospectively, Dias-Neto et al. reported that AAA patients had higher PVAT density around aneurysmal sacs than in healthy areas, correlating with aortic volume [97]. In other findings, periaortic fat correlated with larger aortic dimensions, both thoracically and abdominally, positioning local fat accumulation as a contributor to aortic remodeling [98]. Recent work aimed at assessing the correlation between the radiomic features of PVAT and AAA growth following EVAR suggested PVAT as an independent risk factor for aneurysm dilatation to help clinicians monitor and manage postoperative patients. Disappointingly, only clinical data from male patients were included here [99]. Perivascular wall inflammation was also strongly associated with the risk of re-intervention after EVAR in another study by Gao et al., 2023 [96]. Their cohort, at least, included 25% female patients [100]. Clearly, PVAT may play a role in all pathological stages of AAA formation, predicting the tissue as a useful new target for AAA therapy. Considering the limited number of animal and human studies, further evidence is necessary to unravel the complex nature of the perivascular stroma in acute aortic events.

## 5. Sex-Specific Differences in PVAT

Aside from the differences in anatomical distribution and associated cellular properties, current studies suggest that PVAT is sexually dimorphic [101,102], which may have profound implications for vascular physiology and pathology.

### 5.1. PVAT Morphological Differences

Human fat depots differ between men and women. Compared to men, who typically show hypertrophy of abdominal adipocytes, women generally have greater expansion of subcutaneous adipose tissue, which contributes to a higher number of adipocytes in the gluteo-femoral depots [67,103,104]. The mechanisms linking this fat to metabolic risks are unclear [105]. As fat has a potential relevance to vascular homeostasis and metabolism, the diversity of local periaortic fat is of particular interest in aortic pathology. Owing in particular to changes in visceral fat deposition after menopause [106], it is presumed that the cellular interplay between the perivascular stroma and the arteries also varies according to sex. Females tend to lower PVAT in contrast to male rats. Sex dimorphism may result from gene expression, with more adipocyte progenitors in males [107]. The fact that phenotypic variability in PVAT is not uniform across anatomical locations or sex has been highlighted so far. To identify putative sex disparities in PVAT, it is important to comprehend the initial morphology of the adipocytes surrounding the aorta. Notably, PVAT along the thoracoabdominal aorta differentiates into two locally divergent populations, varying in both morphology and functional characteristics: thoracic (tPVAT) and abdominal (aPVAT) PVAT [108]. tPVAT is characterized by brown adipose tissue (BAT) morphology, with small multilocular adipocytes and abundant mitochondria. In humans, thoracic peri-aortic fat mass is linked to hypertension, diabetes and aortic/coronary calcification when adjusted for body mass index (but not visceral fat) [47]. Additionally, midlife PVAT around the descending aorta may contribute to poorer physical function in older age [109,110,111]. In contrast, the aPVAT contains large unilocular white adipocytes (WAT). Human PVAT mainly consists of WAT. Region-specific PVAT differences exist in lipid composition and gene expression. While aPVAT has both more unsaturated fatty acids [112], and a higher expression of inflammatory cytokines and receptors (e.g., IL-6R, TNFR1/2), tPVAT is characterized by genes predisposing to browning (e.g., UCP-1) [108,113]. In brief, tPVAT tends to have a protective, brownish character, probably contributing to the alleviation of the atherosclerotic plaque burden in mice [114]. Conversely, elevated tPVAT may be associated with adverse cardiometabolic events in humans, as demonstrated by the 2012 Framingham Heart Study, suggesting proatherogenic activity [98]. Furthermore, proinflammatory aPVAT predisposes to aneurysm formation [14]. Interestingly, aPVAT itself has a distinct sex-specific immune status, as males contain more immune cells, particularly T-cells [115,116]. Although female rats had fewer PVAT precursor cells, they had more fat-derived immune cells, underscoring the complexity of PVAT. The same work highlights physiological similarities in the perivascular immune profile compared to other fat depots by characterizing subpopulations of lymphocytes and macrophages [116], as opposed to another study establishing higher pro-inflammatory macrophages in PVAT than in subcutaneous fat [117]. Researchers also evaluated the relationship between perivascular inflammation and plaque vulnerability, finding higher prevalence of thin-cap fibroatheroma and macrophage accumulation in women, but not in men. Knowledge of the underlying causes is still limited by the small sample size of women [118].

Adipocytes are predisposed to aberrant expansion with inflammation, hyperplasia and hypertrophy, leading to ECM remodeling and atypical adipocyte morphology, creating a pathological perivascular niche [47]. A recent study on PVAT function in hypertension, using a preclinical stroke-prone spontaneously hypertensive rat model (SHRSP), suggests that male PVAT may exhibit impaired vasorelaxation, along with larger and less densely distributed adipocytes compared to females [119,120]. These observations align with findings of Contreras et al., who reported larger lipids in male PVAT [121]. It is conceivable that such enlarged fat cells could lead to “overflow” and ectopic fat storage, potentially triggering ROS production, endoplasmic reticulum (ER) stress and inflammation [68,122]. The current hypothesis is that altered adipocyte plasticity is a donor to the pathogenic function of PVAT, possibly in a sex-dependent way. New strategies to improve AA outcomes may be derived from understanding how these morphological and pathological PVAT adaptations mechanistically differ between the sexes.

### 5.2. Sex-Specific Impact on PVAT Vascular Tone Regulation

In line with the aforementioned chapters, PVAT plays a prominent role in the regulation of vascular tone, with dysfunction leading to impaired ECM remodeling and endothelial malfunction, weakening the arterial wall and increasing the risk of atherosclerotic lesions [47]. Emerging research suggests that PVAT functionality differs between sexes, impacting vascular health and disease outcomes. The beneficial effects of PVAT secreting vasoactive molecules are controversially discussed. Namely, male obese rats had reduced thoracic PVAT function, loss of anti-contractile activity and increased fibrosis compared to females [123]. Conversely, females may be relatively protected from these adverse effects. For example, Kogota et al., recently showed that compensation for endothelial dysfunction persists into old age in female PVAT, unlike in males [124]. Obesity, a major risk factor for cardiovascular comorbidities, triggered sex-specific molecular transformation in mesenterial PVAT, with male mice exhibiting marked upregulation of ECM pathways and enhanced collagen deposition. Surprisingly, female mice are not protected from obesity-induced PVAT dysregulation by ovarian hormones [53], implying underlying causes not primarily reliant on hormonal status. Lazaro’s group showed that the hydrophobic glycoprotein cholesteryl ester transfer protein (CETP) exerts sex-specific actions on PVAT, impairing male function through oxidative stress and inflammation, while improving female function by increased NO and anti-inflammation [125,126]. A subsequent hypothesis was proposed by Hanscom et al., who questioned whether neurons innervating PVAT provide adrenergic drive to stimulate murine PVAT adipocytes. Despite being associated with blood vessels, nerve fibers showed limited anatomical and functional interactions with adipocytes, emphasizing an alternative mechanism controlling the adrenergic anticontractile functions of PVAT [127]. Furthermore, Wabel et al., (2024) identified chemerin, produced by PVAT, as a key adipokine controlling vascular tone [122]. In general, chemerin potentiates agonist-induced vasoconstriction (e.g., norepinephrine and serotonin). A significant reduction in aortic pulse wave velocity was measured in chemerin knockout Dahl SS rats, confirming its role in regulating aortic function [128]. Compared to males, females exhibited lower chemerin protein levels in aortic PVAT. Despite this, the main limitation of the study is the use of a global knockout model. Combining a PVAT-specific Cre animal model with a genetic chemerin deletion might be beneficial to further elucidate PVAT-associated mechanisms. Furthermore, stimulation of the adiponectin receptor appears to contribute to the anticontractile effect of porcine coronary artery PVAT in females [129]. The same group showed that PVAT-released prostanoids behave in a sex-specific manner in porcine coronary arteries. PVAT has been validated as a source of potent vasoconstrictors such as thromboxane A2 [106], which is also involved in the progression of atherosclerosis [130]. Thromboxane is required for PVAT-induced contraction in female arteries, whereas PGF2α is sufficient in male arteries [131]. Given the observed sexual dimorphism in PVAT traits, it is imperative to explore novel approaches to elucidate the mechanisms underlying PVAT maladaptation and its role in maintaining vascular tone.

### 5.3. Impact of Sex Hormones on PVAT

Sex hormones have multiple effects on adipose tissue function [132], most notably in the changes that occur during puberty and menopause [105]. Steroid hormones, such as estrogens, particularly 17^2^-estradiol (E2), are key regulators of metabolism by regulating adipocyte gene expression and adipose tissue secretory activity [105,133,134]. Consequently, estrogens modulate obesity-related comorbidities such as insulin resistance, hypertension and endothelial dysfunction, which increase the risk of metabolic and cardiovascular disease [106,134,135]. Thus, it is not surprising that PVAT levels are associated with attenuated insulin sensitivity and diminished regulation of vasoreactivity in humans [136,137]. While the paradigm for studying sex differences in cardiovascular morbidity is largely based on endothelial cell function as the dogma of vascular health [106,138], changes in the directly adjacent PVAT are now becoming the focus of increasing research interest.

Premenopausal women are less likely to suffer from aortic events than men [134,135]. In contrast, postmenopausal estrogen loss favors the accumulation of various types of adipose tissue, including visceral and perivascular fat. Increased adipogenesis in women may lead to a greater predisposition to obesity-related cardiovascular disease than in age-matched men [98,106,111,139,140]. Clinical studies have shown not only an excess of postmenopausal perivascular and pericardial adiposity, but also a positive correlation between aortic PVAT volume and E2 reduction [111,141]. Research utilizing ovariectomized (OVX) animal models has discovered that OVX provokes PVAT dysfunction owing to hypertrophy, inflammation, and MMP9 activation, initiating arterial fiber degeneration and detrimental vascular changes in a sexually dimorphic manner [142]. OXV was proven to attenuate anti-contractile effects and to potentiate the infiltration and proliferation of pro-inflammatory and pro-adipogenic molecules in PVAT [143]. Interestingly, the protective effect observed in female high-fat diet mice against molecular remodeling in the mesenteric PVAT-ECM appears to be independent of ovarian hormones, adding to the complexity associated with PVAT integrity [53]. The role of androgens, whose presence and function in PVAT is poorly studied, is also of relevance. In postmenopausal women, a 4- to 7-fold higher E2 concentration was found in both visceral and subcutaneous adipose tissue after hormone therapy. There was also a higher estrogen/androgen ratio in adipose tissue, indicating a shift towards local estrogen effects [144].

It is not entirely certain how adverse vascular changes, and clinical complications differ between sexes, but this may be due to sexually dimorphic perivascular fat accumulation and associated hormonal changes. There are clear indications of a possible link between sexual hormones, PVAT and the development of AAA: estrogen appears to impact AAA by modulating PVAT and endothelial function. PVAT expansion, especially in postmenopausal women, may exacerbate vascular remodeling and inflammation, contributing to AAA. Hence, a deeper comprehension of perivascular signaling mechanisms is fundamentally required to gain insight into the gender-specific risk factors and therapeutic targets for AAA.

## 6. Quantification of PVAT Inflammation

Computed tomography (CT), which due to its very high spatial resolution is also considered the gold standard for visualizing and characterizing PVAT, is a key tool in medical diagnostics [17]. The structure and composition of PVAT can be determined by quantifying the fat attenuation index (FAI). This has been shown to reflect transcriptomic, metabolic, and phenotypic alterations in PVAT [47]. The FAI was originally used to evaluate coronary inflammation. Indeed, routine coronary CT angiograms (CCTA) can visualize and quantify the gradient of lipid accumulation and adipocyte size around inflamed coronary arteries [17,81]. Building on a recent theory of vascular crosstalk signaling, Antoniades and colleagues proposed an internal “thermometer” of vascular inflammation. In addition to impairing adipogenic differentiation [81], PVAT lipolysis is stimulated, resulting in a gradient of smaller adipocytes close to the local inflamed vascular wall [17].

Important research aims to explore the early detection of vascular inflammation through PVAT changes using CT scans, attempting to link ex vivo images to adipose tissue biology [81]. FAI predicts not only early subclinical coronary disease but also plaque vulnerability, suggesting an early detection marker in the clinical setting [81]. Consistent with these observations by Antonopolous et al. [81], the Oikonomou group in their CRISP CT study confirmed the association of high FAI with cardiac mortality in a post hoc analysis of coronary outcome data from two independent cohorts [145]. In sum, FAI correlates positively with atherosclerotic plaque burden and inversely with adipogenic gene expression [81,140,145]. Kinoshita et al., who investigated the gender-specific associations between PVAT inflammation and plaque vulnerability, determined a higher inflammatory status in women [118]. As mentioned before, menopausal transition is a recognized risk factor for cardiovascular disease [141]. A single-center, retrospective, case-control study analyzed 140 perimenopausal women with and without coronary heart disease and developed an integrated FAI model that showed improved diagnostic accuracy for perimenopausal coronary heart disease [142]. The results emphasize the necessity to continue researching gendered cohorts. In conclusion, a major advance in understanding the role of inflammation in coronary artery disease is the integration of PVAT assessment.

The progression of atherosclerosis may be mediated by bidirectional signaling between the coronary arteries and the adjacent PVAT [59]. Nonetheless, the state of knowledge regarding PVAT function and the underlying mechanisms in aortic and peripheral arteries appears to be incomplete. Meanwhile, perivascular FAI is also coming into focus as a non-invasive biomarker for aortic aneurysms. As described in the previous chapters, PVAT seems to be directly linkable to AAA. Several studies have investigated the relationship between PVAT density and AAA, emphasizing that higher fat density in AAA patients is associated with chronic inflammation [97]. Besides being a marker of origin for estimating coronary fat, the results underline the FAI’s potential for detecting and monitoring AAA.

Given the preliminary nature of the current findings, future studies to validate the prognostic utility of perivascular FAI in different patient populations, with particular attention to gender differences, are urgently recommended. Importantly, perivascular FAI implications for therapeutic paradigms and the integration of advanced imaging into routine care must be considered. With all the significant discoveries to date, several limitations remain. The variability in methodologies and imaging techniques has challenged the use of perivascular FAI as a biomarker. The use of retrospective analyses, relatively small patient cohorts has led to questions about the generalizability and variability in the findings, prompting further validation research in larger prospective trials [17,59,144]. Artificial intelligence and radiomic analysis could be used to improve the assessment of PVAT in health and disease [44,145].

## 7. Conclusions and Further Direction

AAs exhibit gender differences in prevalence, progression and outcome, with women suffering worse outcomes and faster aneurysm growth. Inflammation and PVAT dysfunction predispose to AA, and hormonal and biomechanical factors, particularly in postmenopausal women, exacerbate the condition. Current guidelines do not adequately address these sex dimorphisms, highlighting the need for tailored treatment strategies and further research. In this review, we discussed the rising relevance of PVAT in vascular health, its potential therapeutic implications for AA, and identify gaps in the current state of research.

PVAT, which surrounds the blood vessels, actively regulates vasomotor tone and vascular homeostasis. Dysfunctional PVAT is associated with chronic low-grade inflammation leading to pathological vessel-wall remodeling. Indeed, accumulating preclinical and clinical evidence suggests that the PVAT inflammatory phenotype and respective vascular metabolic profile may play a fundamental role in the pathogenesis of AAs.

Novel imaging techniques, such as non-invasive CT measurement of perivascular FAI as a biomarker, are gaining ground as a perspective of assessing inflammatory aortic changes in routine clinical practice. A causal connection between excessive perivascular adipogenesis and AAA rupture has been demonstrated, suggesting that modulating vascular adipogenesis could prevent aortic events.

PPARγ, an important regulator of adipocyte function [146], plays a role in PVAT-mediated AA development. Reduced PPARγ levels appear to be linked with increased inflammation, which is likely to promote AAA. Therefore, glitazones, including PPARγ agonists, represent a promising strategy to prevent PVAT-mediated vascular disturbances. Non-steroidal anti-inflammatory drugs [47] and antidiabetic agents (e.g., GLP-1 receptor agonists, SGLT2 inhibitors) [17] also show potential cardioprotective effects, although their direct impact on PVAT function is still under investigation. Statins—widely known for their vasodilatory effects—may influence the PVAT phenotype [147], particularly after an acute coronary syndrome [81]. Considering the observed adaptive secretory properties of PVAT, future studies should prioritize the exploration of the molecular signaling pathways in the perivascular space.

Ongoing research is emphasizing sex differences in PVAT, affecting its composition, metabolism and vascular physiology. Adipokines display sex-specific changes in the perivascular anti-contractile properties, underscoring the need for personalized treatment modalities. More broadly, hormonal effects on PVAT are critical in managing the inherent cardiovascular risks faced by men and women. The known immunomodulatory effects of estrogen generally protect women against AAA formation [148]. Yet, post-menopausal estrogen loss may promote PVAT dysfunction and worsen aortic outcomes, highlighting the importance of sex-specific considerations.

With all pivotal discoveries mentioned, the integration of interface concepts and continued technological advancements will be essential to progress the field of PVAT research and translate findings into clinical practice.

## 8. Methods and Limitation

A narrative literature review was conducted using the PubMed database as the primary source of relevant studies. The search strategy included the use of Medical Subject Headings (MeSH) and keyword combinations such as aortic aneurysm, inflammation, gender, sex, sex-specific differences and perivascular adipose tissue. Articles were selected based on their relevance to the research focus, with particular attention to sex-specific pathophysiological mechanisms in aortic aneurysms and the role of inflammation and adipose tissue. The main limitation is that, due to the lack of preclinical and clinical studies on this topic, a narrative review was conducted rather than a systematic review. Given the limited availability of data on certain aspects of the topic, no strict time frame was applied to the literature search to include a wide range of studies. Looking forward, there is an urgent need for more comprehensive studies involving both male and female subjects to fully elucidate the sex-specific responses of PVAT in aortic disease.

## Figures and Tables

**Table 1:** Anatomy and structural composition.

Category	Description	References
Localization and composition	PVAT surrounds large blood vessels and consists of 40–50% adipocytes embedded in fibrous connective tissue, nerves, vasa vasorum, and a variety of stromal-vascular cells including preadipocytes, fibroblasts, endothelial cells, mesenchymal cells, vSMCs, and immune cells	[40,41,42]
Morphological plasticity	PVAT displays features of both white and brown adipose tissue depending on anatomical location. Thoracic PVAT resembles thermogenic brown adipose tissue, abdominal PVAT is more similar to metabolically active white adipose tissue	[41,43,44,45]
Species differences	Human PVAT is predominantly white adipose tissue, whereas rodent studies often describe more brown-like characteristics, highlighting species differences	[41,46]

**Table 2:** Normal and pathological PVAT functioning.

Functional State	Features/Mechanisms	References
Physiological	PVAT exerts vasoprotective and anti-inflammatory effects secretion of adipokines (e.g., adiponectin, leptin) Vascular tone regulation (e.g., via ADRF and PDRF), contribution to endothelial function via IL-10 and nitric oxide (NO)	[41,47,48,49,55,56]
Pathological (e.g., obesity, atherosclerosis)	Metabolic imbalances lead to PVAT-dysfunction with increased vasoconstriction and inflammation Altered adipokine secretion (↑ leptin, IL-6, TNF-α; ↓ adiponectin, NO, H_2_S) promotes ROS production, oxidative stress, and vascular damage	[17,41,42,46,55,59,60,61,62,63]
Atherogenesis (plaque formation)	PVAT-derived adipokines act via outside-in signaling, promoting LDL accumulation, vSMC migration, foam cell formation, and plaque vulnerability through reduced collagen synthesis and fibrous cap thinning	[41]
Immune modulation	A balance between pro-inflammatory (TNF-α, IL-6) and anti-inflammatory (IL-10) cytokines is crucial Pathological PVAT ↑ CD68^+^ macrophages and CD4^+^ T-cells, contributing to chronic inflammation and impaired vasodilation	[49,50,51,52]

## Data Availability

Not applicable.
